# 

**Published:** 1898-01-29

**Authors:** 


					I the hospital. "The standard of highesl purity ."?Lancet. Jan. 29th, 1898.
CADBURY'S U fea CADBURY'S
COCOA HI jm M COCOA
ABSOLUTELY PURE. DRUGS OR ALKALIES USED, : i
RWICH HOSPI TAL
No. 592. Vol. XXIII. Saturday,
Jan. 29th, 1898.
[ sto p,?506. J PB.ICE TWOPENCE.

				

